# Association analysis of *ANK3* gene variants with schizophrenia in a northern Chinese Han population

**DOI:** 10.18632/oncotarget.13043

**Published:** 2016-11-03

**Authors:** Xiaojuan Guo, Yani Zhang, Jieli Du, Hua Yang, Yini Ma, Jingjie Li, Mengdan Yan, Tianbo Jin, Xianyang Liu

**Affiliations:** ^1^ Xi'an Mental Health Center, Xi’an, Shaanxi 710061, China; ^2^ Inner Mongolia Medical University Hohhot 010010, Inner Mongolia, China; ^3^ School of Life Sciences, Northwest University, Xi’an 710069, China; ^4^ Key Laboratory of Molecular Mechanism and Intervention Research for Plateau Diseases of Tibet Autonomous Region, School of Medicine, Xizang Minzu University, Xianyang, Shaanxi 712082, China; ^5^ Key Laboratory of High Altitude Environment and Genes Related to Diseases of Tibet Autonomous Region, School of Medicine, Xizang Minzu University, Xianyang, Shaanxi 712082, China; ^6^ Key Laboratory for Basic Life Science Research of Tibet Autonomous Region, School of Medicine, Xizang Minzu University, Xianyang, Shaanxi 712082, China

**Keywords:** ANK3, schizophrenia risk, association, case-control study, Chinese Han population

## Abstract

Schizophrenia is a chronic, severely debilitating mental disorder. Many studies have suggested that genetic factors play an important role in the onset and development of schizophrenia. In our study, we conducted a case-control study in a northern Chinese Han population of 499 schizophrenia patients and 500 controls to investigate the effect of variant genotypes of 13 SNPs in ANK3 on schizophrenia risk. Odds ratios (OR) and 95% confidence intervals (CI) were estimated using the chi-squared test, genetic model analysis, and haplotype analysis. Four ANK3 SNPs were associated with schizophrenia risk. The minor allele of rs958852 in ANK3 was associated with a 0.75-fold reduction in schizophrenia risk in an allelic model. In the genetic model, rs958852 was associated with a reduced schizophrenia risk, and rs10994336, rs10994338 and rs4948418 were associated with an increased schizophrenia risk (rs10994336, OR = 2.00, 95%CI: 1.01–3.94, *p* = 0.047; rs10994338, OR = 1.99, 95%CI: 1.01–3.93, *p* = 0.047; rs4948418, OR = 2.00, 95%CI: 1.01–3.94, *p* = 0.047). In addition, haplotype “TTC” of ANK3 was associated with a 0.73-fold reduced schizophrenia risk (95%CI: 0.54–0.99; *p* = 0.044). To our knowledge, this is the first to report of an association between ANK3 rs10994336, rs10994338, rs4948418 and rs958852 and schizophrenia risk in a northern Chinese Han population.

## INTRODUCTION

Schizophrenia is a chronic, severely debilitating mental disorder. Approximately 1% of the population suffers the torment of schizophrenia [1], and it is reportedly the 8th leading cause of disability among people aged 15 to 44 years, worldwide [2]. The exact pathogenesis of schizophrenia remains unknown and, despite large numbers of trials of potential therapies, the efficacy of pharmacological treatments is poor for many schizophrenia patients [3]. Although the gender ratio among patients is nearly equal, females tend to have a later onset than males, and an earlier onset in females almost associated with a family history of schizophrenia [4]. Lichtenstein et al estimated the heritability of schizophrenia to be 64% [5].

A variety of studies have suggested that genetic factors play a key role in the onset and development of schizophrenia. In genome-wide association studies (GWAS), for example, around 30 loci have been identified as being associated with schizophrenia [6]. In addition, an association between *ANK3* and schizophrenia has been detected in several different ethnic groups. The rs10761482 variant is reportedly associated with schizophrenia risk in Norwegian subjects [7], but not in subjects from Germany [8] or southern China [9]. On the other hand, the rs10994336 variant is associated with bipolar disorder in Malays [10] and southern Chinese [9], whereas rs10994336 was found not to be associated with schizophrenia in cohorts of German and Nordic subjects [8, 11].

The associations between *ANK3* polymorphisms and schizophrenia risk have not been investigated in the northern Chinese Han population. In present study, therefore, we selected 13 *ANK3* SNPs whose association with schizophrenia or bipolar disorder has been previously investigated [7, 10–21] and evaluated whether these polymorphisms are associated with schizophrenia risk in a northern Chinese Han population.

## RESULTS

A total of 499 schizophrenia cases and 500 unrelated healthy controls were enrolled in our study. The demographic data for the cases and controls are shown in Table 1. The mean ages were 67.11 ± 9.24 years and 64.31 ± 8.91 years for the case and control groups, respectively. As shown in Table 2, 13 SNPs were analyzed, and two (rs10761482 and rs3808942) displayed significant deviation from the Hardy-Weinberg equilibrium (*p* < 0.05); We used the Pearson Chi-squared test to compare the different allele frequency distributions between the cases and controls and found that rs958852 was significantly associated with a 0.75-fold reduction in the risk of schizophrenia (95%CI 0.58–0.97; *p* = 0.025) (Table 2).

**Table 1:** Characteristics of cases and controls in this study *n* (%)

Variable	Case	Control	*p*value
Sex			< 0.001^a^
Male	263 (52.7)	192 (38.4)	
Female	236 (47.3)	308 (61.6)	
Age, year (mean ± SD)	36.68 ± 13.25	50.35 ± 7.76	< 0.001^b^

*p* ≤ 0.05 indicates statistical significance.

a Two-sided Chi-squared test.

b Independent samples *t* test.

**Table 2:** Candidate SNPs examined in ANK3 (10q21.2) in cases and controls and odds ratio estimates for schizophrenia

SNP No.	Alleles A/B	MAF (case)	MAF (control)	*p*^a^value for HWE test	OR(95% CI)		*p*^b^adj.
rs12761450	T/G	0.048	0.055	0.053	0.87	0.58–1.29	0.485
rs10761482	T/C	0.273	0.260	0.007*	1.07	0.87–1.30	0.526
rs3808942	C/T	0.230	0.232	0.032*	0.99	0.81–1.22	0.935
rs10994336	T/C	0.229	0.232	0.102	0.99	0.80–1.21	0.893
rs10994338	A/G	0.227	0.231	0.102	0.98	0.79–1.20	0.831
rs4948418	T/C	0.230	0.232	0.102	0.99	0.81–1.22	0.935
rs10994359	C/T	0.334	0.328	0.362	1.03	0.85–1.24	0.788
rs10994397	T/C	0.329	0.320	0.682	1.04	0.86–1.25	0.679
rs1938526	G/A	0.341	0.320	0.679	1.10	0.91–1.33	0.308
rs10994415	C/T	0.349	0.320	0.838	1.14	0.94–1.37	0.174
rs958852	T/A	0.123	0.158	0.313	0.75	0.58–0.97	0.025*
rs16915157	T/C	0.375	0.346	0.490	1.13	0.94–1.36	0.181
rs1837950	T/C	0.416	0.415	0.927	1.00	0.84–1.20	0.970

SNP single nucleotide polymorphism, HWE Hardy-Weinberg equilibrium, OR odds ratio, 95% CI 95% confidence interval, MAF minor allele frequency

* *p* ≤ 0.05 indicates statistical significance

a *p* was calculated by exact test

b *p* was calculated by Pearson Chi-squared test

We also assumed that the minor SNP allele was a greater risk factor than the wild-type allele (Table 3). We used three genetic models (dominant, recessive and additive) to analyze the associations between the SNPs and the schizophrenia risk. We found that rs10994336, rs10994338 and rs4948418 were associated with schizophrenia risk in the recessive model (OR 2.00; 95%CI 1.01–3,94; *p* = 0.047, OR 1.99; 95%CI 1.01–3.93; *p* = 0.047 and OR 2.00; 95%CI 1.01–3,94; *p* = 0.047, respectively). In addition, rs958852 was associated with schizophrenia risk in the dominant (OR 0.69; 95%CI 0.49–0.97; *p* = 0.031) and additive (OR 0.73; 95%CI 0.54–0.99; *p* = 0.044) models.

**Table 3:** Logistic regression analysis of the association between SNPs and schizophrenia risk *n* (%)

SNP No.	Minor	Dominant model			Recessive model			Additive model		
	allele	OR	95% CI	*P*^a^	OR	95% CI	*P*^a^	OR	95% CI	*P*^a^
rs10994336	T	0.91	0.68–1.23	0.555	2.00	1.01–3.94	0.047*	1.03	0.80–1.33	0.799
rs10994338	A	0.90	0.67–1.21	0.487	1.99	1.01–3.93	0.047*	1.02	0.80–1.31	0.868
rs4948418	T	0.92	0.68–1.24	0.577	2.00	1.01–3.94	0.047*	1.04	0.81–1.33	0.779
rs958852	T	0.69	0.49–0.97	0.031*	0.87	0.28–2.66	0.802	0.73	0.54–0.99	0.044*

* *p* ≤ 0.05 indicates statistical significance

a *p* values were calculated by unconditional logistic regression adjusted for age and gender

Comparisons of the SNP genotypes and their associations with the risk of schizophrenia are shown in Table 4. We found that genotype “TA” of rs958852 in *ANK3* was associated with a 0.68-fold lower risk of schizophrenia than genotype “AA” at the 5% level (95%CI 0.48–0.97; *p* = 0.031).

**Table 4:** Comparisons of genotypes of some special SNPs and their associations with schizophrenia risk

SNP ID	Test	OR	95% CI	*p*
rs10994336	TT/CC	1.86	0.93–3.70	0.080
	TC/CC	0.83	0.60–1.13	0.234
	(TT+TC)/CC	–	– –	0.069
rs10994338	AA/GG	1.84	0.92–3.67	0.084
	AG/GG	0.81	0.59–1.11	0.193
	(AA+AG)/GG	–	– –	0.060
rs4948418	TT/CC	1.86	0.93–3.71	0.079
	TC/CC	0.83	0.61–1.14	0.248
	(TT+TC)/CC	–	–	0.071
rs958852	TT/AA	0.79	0.26–2.43	0.678
	TA/AA	0.68	0.48–0.97	0.031*
	(TT+TA)/AA	–	– –	0.094

* *p* ≤ 0.05 indicates statistical significance.

*p* values were calculated from Wald's test adjusted by age and gender

**Figure 1:**
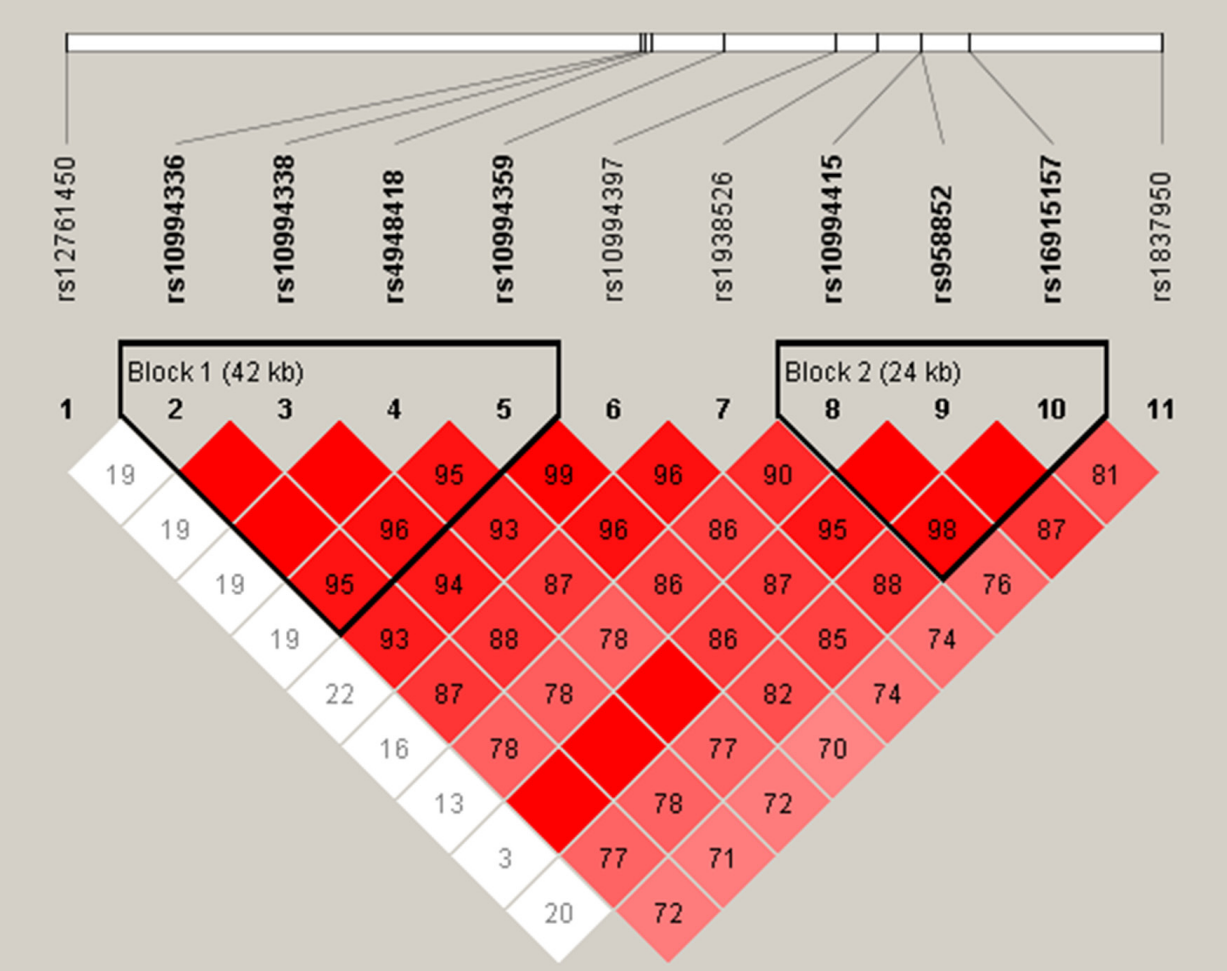
Haplotype block map for all the *ANK3* SNPs Linkage disequilibrium (LD) plots containing eleven SNPs from 10q21.2 of ANK3. Red squares indicate statistically significant associations between a pair of SNPs, as measured by D’; darker shades of red indicate higher D’ values.

We next used the genotype data from all the subjects to do an LD analysis. Two blocks were detected based on haplotype analysis. One block included four SNPs (rs10994336, rs10994338, rs4948418 and rs10994359) with D’ = 0.95, and the other included three SNPs (rs10994415, rs958852 and rs16915157) with D’ = 0.98 (Figure 1). The associations between the *ANK3* haplotypes and the schizophrenia risk are listed in Table 5. We found the “TTC” haplotype was associated with a reduced risk of schizophrenia (OR 0.73; 95%CI 0.54–0.99; *p* = 0.044).

**Table 5:** The haplotypes of three SNPs(rs10994415, rs958852 and rs16915157) and risk of schizophrenia(adjusted by age and gender)

Haplotype	Freq(case)	Freq(control)	Chi-square	*p*^a^	OR	95% CI		*p*^b^
CAT	0.347	0.317	1.986	0.159	1.11	0.89	1.39	0.347
TAT	0.028	0.029	0.016	0.899	1.02	0.55	1.89	0.962
TTC	0.123	0.158	4.992	0.025*	0.73	0.54	0.99	0.044*
TAC	0.500	0.493	0.098	0.754	1.06	0.86	1.30	0.585

* *p* ≤ 0.05 indicates statistical significance.

a *p* value from were calculated from two-sided Chi-squared test.

b *p* values were calculated by unconditional logistic regression adjusted for age and gender.

Lastly, we looked for interactions among the 13 SNPs tested with schizophrenia risk stratified based on gender (Supplementary Table S1). We found that none of the analyzed SNPs correlated with gender had an impact on the association with schizophrenia risk (data was shown in supplementary).

## DISCUSSION

In this case-control study, we investigated associations between genetic variations in *ANK3* and schizophrenia risk in a relatively large sample of individuals. Four susceptibility loci from *ANK3* (rs10994336, rs10994338, rs4948418 and rs958852) were significantly associated with schizophrenia risk in a northern Chinese Han population for the first time. We also found that the “TTC” haplotype of *ANK3* is associated with a 0.73-fold reduction in the risk of schizophrenia.

Many earlier studies focused on gene-related mechanisms or other potential causes for schizophrenia. Okazaki et al [22] suggested that an imbalance in the immune system contributes to the pathophysiology of schizophrenia. Brown et al reported that cardiovascular disease is the most important natural cause of death among schizophrenia patients [23, 24]. Hjelm et al suggested that the genetic etiology of schizophrenia must be strikingly complex, as they were unable to identify any special gene associated with schizophrenia [25]. Several studies have reported that *ANK3* plays an important role in the pathogenesis of schizophrenia. ANK3 is reportedly a neurodevelopmental gene [26] that encodes ankyrin-G (ANKG), which is important for the stability of the neuronal membrane [27]. Notably, the neurodevelopment hypothesis has become the mainstream hypothesis of the etiology of schizophrenia [28]. What's more, ANKG associates with Nav1.5 and recruits the channel to the myocyte membrane, and impaired Nav1.5 function caused by low ANKG levels leads to sinus node dysfunction, conduction defects, and ventricular arrhythmia [29]. This is consistent with the earlier report that cardiovascular disease is a frequent cause of death among schizophrenia patients. At the same time, *ANK3* was found to be associated with bipolar disorder in the large collaborative GWAS [30], and Cassidy et al suggested that schizophrenia and bipolar disorder share common pathogenic pathways [13], as evidenced by the genetic overlap between schizophrenia and bipolar disorder [31].

Rs10994336 was identified as a significant susceptibility locus for bipolar disorder in study of 4,387 cases and 6,209 controls [32], and in a mate analysis [33]. One UK study tried to investigate whether the SNP loci significant in bipolar disorder are also significant in schizophrenia, but they failed to demonstrate a significant association between rs10994336 and schizophrenia risk. In the present study, we observed that rs10994336 is associated with an increased risk of schizophrenia in a northern Chinese Han population. We suppose the difference between our findings and those of the UK study reflects the difference in the ethnic groups.

Associations between rs10994338 and rs4948418 and bipolar disorder have also been previously reported [33, 34]. In present study, we found that rs10994338 and rs4948418 were associated with an increased schizophrenia risk in the northern Chinese Han population. We also found a new significant SNP, rs958852, which appears to reduce the risk of schizophrenia. To our knowledge, this is the first report of an association between ANK3 rs10994336, rs10994338, rs4948418 and rs958852 and schizophrenia risk in a northern Chinese Han population. Our findings suggest *ANK3* may play an important role in schizophrenia. It is anticipated that future studies will clarify the precise contribution of *ANK3* to schizophrenia, which may offer a new strategies for the prevention and treatment of schizophrenia.

Although there are important discoveries revealed in our study, there are also some potential limitations. First, we selected SNPs with *p* values higher than 5% in the HWE test. This approach may exclude some SNPs reported to be significantly associated with schizophrenia in previous studies. Second, our study lacks biological function experiments, which will be crucial for elucidating the role of *ANK3* in schizophrenia.

## MATERIALS AND METHODS

### Study participants

We recruited a total of 499 (263 males, 236 females) northern Han Chinese patients diagnosed with schizophrenia between 2011 and 2015. All the subjects were treated at the Xi’an Mental Health Center. All patients were diagnosed and histologically confirmed to suffer from schizophrenia based on the criteria from the DSM-IV (Diagnostic and Statistical Manual of Mental Disorders, the fourth version). The patients had no history of cancer, nephropathy, infection, or other related disease. In addition, to serve as a control sample, 500 (192 male, 308 female) healthy unrelated individuals were recruited from among the Han Chinese living in and around Xi’an city. All of the chosen controls were from the Medical Center in the Affiliated Hospital of Tibet University for Nationalities.

### Clinical data and demographic

The study protocol was approved by the Human Research Committee of the Xi’an Mental Health Center for Approval of Research Involving Human Subjects. Informed consent was obtained from all subjects. All subjects were interviewed by a nurse who collected detailed information about gender, age, region, ethnicity, smoking status, education status, working environment, pressures experienced, family history, and other lifestyle factors.

### SNP selection and genotyping

In this case-control study, we selected 13 SNPs in *ANK3*, each with a minor allele frequency (MAF) higher than 5% in the Han Chinese population. We used the GoldMag extraction method to extract genomic DNA from whole blood (GoldMag Co. Ltd, Xi’an, China) [35]. Spectrometry (DU530UV/VIS spectrophotometer, Beckman Instruments, Fullerton, CA, USA) was used to measure the DNA concentration. Multiplexed SNP MassEXTEND assays were designed using Sequenom MassARRAY Assay Design 3.0 software, and the Sequenom MassARRAY RS1000 recommended by the manufacturer was utilized to perform the SNP genotyping [36]. Genotype data were managed and analyzed using Sequenom Typer 4.0 software [36–38].

### Statistical analyses

Statistical analyses were performed using the SPSS version 16.0 statistical package (SPSS, Chicago, IL, USA) and Microsoft Excel. All *p* values were two-sided, and values of *p* ≤ 0.05 were considered significant. The genotype frequencies for each SNP in the control subjects were checked using the Hardy-Weinberg equilibrium (HWE). Chi-squared test or Fisher's exact test was used to calculate the allele and genotype frequencies among cases and controls. Associations between the genotypes and schizophrenia risk were estimated by computing odds ratios (ORs) and 95% confidence intervals (CIs) evaluated in three genetic models (dominant, recessive, additive model) using unconditional logistic regression adjusted for age and gender. We determined *p* values for trend by entering the variable as a single term in the model (i.e., one degree of-freedom) and testing using Wald's test. Finally, the Haploview software package (version 4.2) and SHEsis software platform (http://analysis.bio-x.cn/myAnalysis.php) were used for estimate the pairwise linkage disequilibrium (LD), haplotype construction, and genetic association at polymorphism loci.

## CONCLUSIONS

In sum, we confirmed a positive association between *ANK3* and schizophrenia and also demonstrated that four *ANK3* variants (rs10994336, rs10994338, rs4948418 and rs958852) are associated with schizophrenia in a northern Chinese Han population. Further evidence will be needed to confirm this interaction in a wider population, and to determine the role of *ANK3* in the development of schizophrenia.

## SUPPLEMENTARY MATERIALS TABLE


