# Supplementary material for: Association analysis of ANK3 gene variants with schizophrenia in a northern Chinese Han population
Source: Oncotarget. 2016 Nov 3;7(52):85888–94. doi: 10.18632/oncotarget.13043 (PMC5349882; doi:10.18632/oncotarget.13043)
Supplement: Supplementary file 1 [file oncotarget-07-85888-s001.pdf]

Association analysis of ANK3 gene variants with schizophrenia in a northern Chinese Han population

Supplementary Materials

Supplementary Table S1: Logistic regression analysis of the association between SNPs and schizophrenia risk stratified by gender

		female			male		
		OR	95% CI	*p*	OR	95% CI	*p*
rs12761450	Dominant model	1.04	0.53–2.04	0.901	1.06	0.49–2.28	0.878
	Recessive model	0.00	0.00– ——	0.999	0.00	0.00– ——	0.999
	Additive model	0.97	0.51–1.84	0.925	0.91	0.46–1.83	0.800
rs10761482	Dominant model	1.06	0.72–1.55	0.780	0.96	0.60–1.53	0.849
	Recessive model	0.73	0.33–1.61	0.435	11.13	1.84–67.31	0.009
	Additive model	0.99	0.72–1.35	0.931	1.18	0.78–1.78	0.444
rs3808942	Dominant model	0.77	0.52–1.14	0.198	0.81	0.50–1.31	0.394
	Recessive model	0.72	0.30–1.75	0.466	3.20	0.86–11.97	0.084
	Additive model	0.80	0.58–1.11	0.179	0.97	0.65–1.47	0.896
rs10994336	Dominant model	0.76	0.52–1.13	0.177	1.16	0.72–1.87	0.550
	Recessive model	1.81	0.79–4.19	0.164	2.10	0.65–6.72	0.214
	Additive model	0.91	0.66–1.25	0.551	1.22	0.82–1.82	0.337
rs10994338	Dominant model	0.77	0.52–1.13	0.181	1.10	0.68–1.78	0.690
	Recessive model	1.81	0.78–4.17	0.166	2.10	0.65–6.72	0.214
	Additive model	0.91	0.66–1.25	0.556	1.18	0.79–1.76	0.426
rs4948418	Dominant model	0.77	0.52–1.14	0.194	1.16	0.72–1.87	0.550
	Recessive model	1.81	0.79–4.19	0.164	2.10	0.65–6.72	0.214
	Additive model	0.91	0.66–1.26	0.579	1.22	0.82–1.82	0.337
rs10994359	Dominant model	0.82	0.56–1.21	0.326	1.13	0.71–1.80	0.612
	Recessive model	0.98	0.54–1.78	0.955	1.62	0.73–3.57	0.235
	Additive model	0.90	0.67–1.19	0.453	1.18	0.83–1.69	0.352
rs10994397	Dominant model	0.76	0.52–1.12	0.170	1.33	0.83–2.12	0.240
	Recessive model	1.00	0.55–1.84	0.989	1.45	0.67–3.16	0.350
	Additive model	0.86	0.65–1.15	0.313	1.27	0.89–1.81	0.189
rs1938526	Dominant model	0.89	0.61–1.32	0.566	1.29	0.81–2.07	0.288
	Recessive model	0.99	0.53–1.82	0.963	1.28	0.59–2.77	0.527
	Additive model	0.94	0.70–1.25	0.653	1.22	0.85–1.74	0.276
rs10994415	Dominant model	0.98	0.67–1.45	0.933	1.36	0.85–2.17	0.203
	Recessive model	0.90	0.49–1.64	0.728	1.66	0.77–3.56	0.195
	Additive model	0.97	0.73–1.29	0.819	1.32	0.93–1.88	0.120
rs958852	Dominant model	0.75	0.48–1.16	0.197	0.59	0.35–1.02	0.057
	Recessive model	0.31	0.06–1.49	0.144	4.29	0.67–27.64	0.125
	Additive model	0.73	0.49–1.08	0.117	0.72	0.45–1.18	0.193
rs16915157	Dominant model	0.94	0.64–1.39	0.755	1.28	0.80–2.05	0.308
	Recessive model	1.10	0.62–1.93	0.754	1.80	0.89–3.67	0.104
	Additive model	0.99	0.75–1.31	0.945	1.31	0.93–1.85	0.124
rs1837950	Dominant model	0.69	0.46–1.04	0.074	1.43	0.88–2.31	0.153
	Recessive model	0.91	0.55–1.51	0.708	1.35	0.72–2.51	0.352
	Additive model	0.82	0.62–1.08	0.156	1.28	0.92–1.79	0.142

*$p \leq 0.05$ indicates statistical significance.
p values were calculated by unconditional logistic regression adjusted for age and gender.
